# The large‐scale structural connectome of task‐specific focal dystonia

**DOI:** 10.1002/hbm.25012

**Published:** 2020-04-20

**Authors:** Sandra Hanekamp, Kristina Simonyan

**Affiliations:** ^1^ Department of Otolaryngology—Head and Neck Surgery Massachusetts Eye and Ear Infirmary Boston Massachusetts USA; ^2^ Department of Otolaryngology—Head and Neck Surgery Harvard Medical School Boston Massachusetts USA; ^3^ Department of Neurology Massachusetts General Hospital Boston Massachusetts USA

**Keywords:** DWI, focal hand dystonia, laryngeal dystonia, motor control

## Abstract

The emerging view of dystonia is that of a large‐scale functional network disorder, in which the communication is disrupted between sensorimotor cortical areas, basal ganglia, thalamus, and cerebellum. The structural underpinnings of functional alterations in dystonia are, however, poorly understood. Notably, it is unclear whether structural changes form a larger‐scale dystonic network or rather remain focal to isolated brain regions, merely underlying their functional abnormalities. Using diffusion‐weighted imaging and graph theoretical analysis, we examined inter‐regional white matter connectivity of the whole‐brain structural network in two different forms of task‐specific focal dystonia, writer's cramp and laryngeal dystonia, compared to healthy individuals. We show that, in addition to profoundly altered functional network in focal dystonia, its structural connectome is characterized by large‐scale aberrations due to abnormal transfer of prefrontal and parietal nodes between neural communities and the reorganization of normal hub architecture, commonly involving the insula and superior frontal gyrus in patients compared to controls. Other prominent common changes involved the basal ganglia, parietal and cingulate cortical regions, whereas premotor and occipital abnormalities distinctly characterized the two forms of dystonia. We propose a revised pathophysiological model of focal dystonia as a disorder of both functional and structural connectomes, where dystonia form‐specific abnormalities underlie the divergent mechanisms in the development of distinct clinical symptomatology. These findings may guide the development of novel therapeutic strategies directed at targeted neuromodulation of pathophysiological brain regions for the restoration of their structural and functional connectivity.

## Graphical abstract

The study by Hanekamp and Simonyan defined the large‐scale structural connectome of task‐specific focal dystonia. It delineated global and nodal alterations that both unify and distinguish between different clinical forms of this disorder. These findings are significant for understanding the pathophysiology of task‐specific focal dystonia and the potential development of novel approaches for therapeutic neuromodulation in these patients.

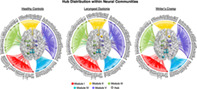

## 1 INTRODUCTION

Isolated dystonia is an umbrella diagnosis for a group of movement disorders characterized by sustained or intermittent muscle contractions leading to involuntary postures or repetitive movements. Focal dystonias affect isolated muscle groups and represent the most common clinical phenotype of this disorder. Among these, task‐specific focal dystonias (TSFDs) impair voluntary behaviors that are associated with precise and highly coordinated motor task performance, such as writing in writer's cramp (WC) and speaking in laryngeal dystonia (LD). Task specificity in dystonia is a clinically well‐documented phenomenon, with symptoms often causing long‐term psychological stress, psychiatric comorbidities, social isolation, and professional disability.

Although the exact pathophysiology of focal dystonia, including TSFDs, is unclear, it is currently being viewed as a disorder of the large‐scale functional connectome (e.g., Battistella, Termsarasab, Ramdhani, Fuertinger, & Simonyan, 2017; Conte et al., 2019; Fuertinger & Simonyan, 2017; Fuertinger & Simonyan, 2018; Neychev, Gross, Lehericy, Hess, & Jinnah, 2011; Schirinzi, Sciamanna, Mercuri, & Pisani, 2018; Simonyan, 2018; Zoons, Booij, Nederveen, Dijk, & Tijssen, 2011). The common, unifying features of the functional connectomes in different forms of focal dystonia include disorganization of the basal ganglia‐thalamo‐cortical community, abnormal distribution of influential regions of information transfer (hubs) in sensorimotor regions and thalamus, and reduced connectivity within the sensorimotor and frontoparietal regions. Moreover, a greater extent of functional alterations involving sensorimotor and executive cortical regions vs. subcortical structures are distinct features of TSFDs, such as LD and WC, compared to non‐task‐specific dystonias, such as cervical dystonia and blepharospasm (Battistella et al., 2017). Different forms of TSFDs are further characterized by abnormalities in the functional specialization of network hubs that are responsible for the various levels of sensorimotor and executive control during production of affected motor behaviors (Fuertinger & Simonyan, 2018).

Despite these advances in identifying functional network properties in TSFDs, their structural underpinnings are less well understood. Microstructural alterations have been reported as gray matter volumetric and cortical thickness changes in the basal ganglia, thalamus, cerebellum, sensorimotor and parietal cortex, as well as white matter aberrations along the cortico‐striato‐pallido‐thalamic and cerebello‐thalamo‐cortical pathways (e.g., Bianchi et al., 2017; Bianchi, Fuertinger, Huddleston, Frucht, & Simonyan, 2019; Delmaire et al., 2007; Delmaire et al., 2009; Garraux et al., 2004; Granert et al., 2011; Ramdhani et al., 2014; Simonyan et al., 2008; Simonyan & Ludlow, 2012). Moreover, significant relationships have been established between abnormalities in brain activation and gray matter structural organization of the primary somatosensory, superior temporal, inferior frontal cortical regions, and cerebellum (Simonyan & Ludlow, 2012), pointing to multi‐domain structure–functional interactions underlying the TSFD pathophysiology. However, it remains unknown whether these structural changes represent abnormal nodes of the large‐scale structural dystonic network or rather have only local impact by underlying regional functional abnormalities. If former, it is critical to establish how structural network alterations are further specialized to contribute to distinct clinical symptomatology of different forms of TSFD.

In this study, we used diffusion‐weighted imaging (DWI) and graph theoretical analysis to identify the overall architecture of the large‐scale structural network and determine its common and distinct alterations in two clinically different forms of TSFD – WC and LD, compared to healthy individuals. We hypothesized that the large‐scale structural connectome is altered in focal dystonia, and its abnormalities closely follow those of the functional connectome. In particular, we postulated that structural network abnormalities commonly involve subcortical structures, such as the basal ganglia and cerebellum, in both forms of TSFD, whereas distinct nodal changes within sensorimotor and executive cortical regions represent specialized abnormalities of the fine motor control that is impaired in each TSFD form.

## 2 MATERIALS AND METHODS

### 2.1 Study subjects

A total of 48 subjects participated in the study, including 17 patients with LD (10 females/7 males, mean age 56.6 ± 13.2 years), 15 patients with WC (9 females/6 males, mean age 53.7 ± 11.7 years), and 16 healthy controls (10 females/6 males, mean age 53.4 ± 12.2 years) (Table 1). All subjects were monolingual native English speakers, right‐handed as determined by the Edinburgh Handedness Inventory, and had normal cognitive status as determined by the Mini‐Mental State Examination. None were carriers of verified dystonia gene mutations, including DYT1, DYT6, DYT4, and DYT25.

**TABLE 1:** Demographics of participants

	Healthy controls	Laryngeal dystonia	Writer's cramp	*p*‐value
	*n* = 16	*n* = 17	*n* = 15	
Age (years; mean ± *SD*)	55.3 ± 11.3	56.6 ± 13.2	53.7 ± 11.7	*p* ≥ .71
Sex (F:M)	10:6	10:7	9:6	*p* ≥ .48
Age of onset (years; mean ± *SD*)	n/a	37.4 ± 12.6	37.7 ± 10.3	*p* ≥ .95
Dystonia duration (years; mean ± *SD*)	n/a	19.2 ± 9.7	16.1 ± 10.7	*p* ≥ .39
BFM movement scale (mean ± *SD*)	n/a	3.6 ± 2.5	4.3 ± 2.6	*p* ≥ .26
BFM disability scale (mean ± *SD*)	n/a	2.1 ± 0.9	1.7 ± 0.8	*p* ≥ .21
Handedness	Right			
Genetic status	Negative for DYT1, DYT6, DYT4, and DYT25			
Cognitive status	Mini‐mental state examination ≥27 points			

Abbreviations: BFM, Burke–Fahn–Marsden Dystonia Rating scale; F, female; M, male; n/a, not applicable.

Dystonia diagnosis was established based on the medical history, neurological and laryngological evaluations, as relevant. WC was focal to the right hand, and LD was focal to the larynx (3 abductor/14 adductor forms). Patients did not have any other neurological (including other forms of dystonia or mirror dystonia in WC), psychiatric, or laryngeal problems. All patients were fully symptomatic at the time of study participation. Those who received botulinum toxin injections were enrolled at least 3 months after their last injection, when fully symptomatic. None were on any medications affecting the central nervous system.

The duration of disorder was 19.2 ± 9.7 years in LD patients and 16.1 ± 10.7 years in WC patients. The age at symptom onset was 37.4 ± 12.6 years in LD patients and 37.7 ± 10.3 years in WC patients. Based on the Burke‐Fahn‐Marsden Dystonia Rating Scale, the symptom severity was 3.6 ± 2.5 on the Movement Scale and 2.1 ± 0.9 on the Disability Scale in LD patients and 4.3 ± 2.6 on the Movement Scale and 1.7 ± 0.8 on the Disability Scale in WC patients. There were no significant differences (*p* ≥ .21) in age, sex, dystonia duration, onset, or severity between the groups based on two‐sample *t* tests or Fisher's exact tests, as appropriate (Table 1).

All participants gave their informed written consent before study participation, which was approved by the Internal Review Board of Partners HealthCare Research Program. Data from some participants were used in our previous studies, which examined regional alterations in gray matter organization, white matter integrity, and resting‐state functional connectivity (Bianchi et al., 2019; Fuertinger & Simonyan, 2018).

## 3 EXPERIMENTAL DESIGN

### 3.1 Image acquisition

A whole‐brain high‐resolution MRI was performed on a 3.0 T Siemens Skyra scanner with a 32‐channel head coil. Uniform T1‐weighted images were acquired using a 3D magnetization prepared rapid acquisition gradient echo sequence with an inversion recovery (3D‐MP2RAGE: TR = 4,000.0 ms, TE = 1.9 ms, TI1/TI2 = 633/1,860 ms, FA = 4°, FOV = 186 × 162 mm, 224 slices, voxel size 1.0 mm^3^, number of averages = 2). A whole‐brain DWI was acquired with anterior–posterior (AP) and posterior–anterior (PA) phase encoding directions, each with 64 noncollinear directions and 6 nondiffusion images (*b*_0_) (TR = 3,880 ms, TE = 90 ms, FA = 80°, FOV = 240 mm, voxel size 2.0 mm3, *b* = 1,000 s/mm2, 69 slices). The subject's head was tightly cushioned and padded to minimize the head movements during scanning; subjects were instructed to remain motionless throughout the scan.

### 3.2 Image preprocessing

**FIGURE 1:**
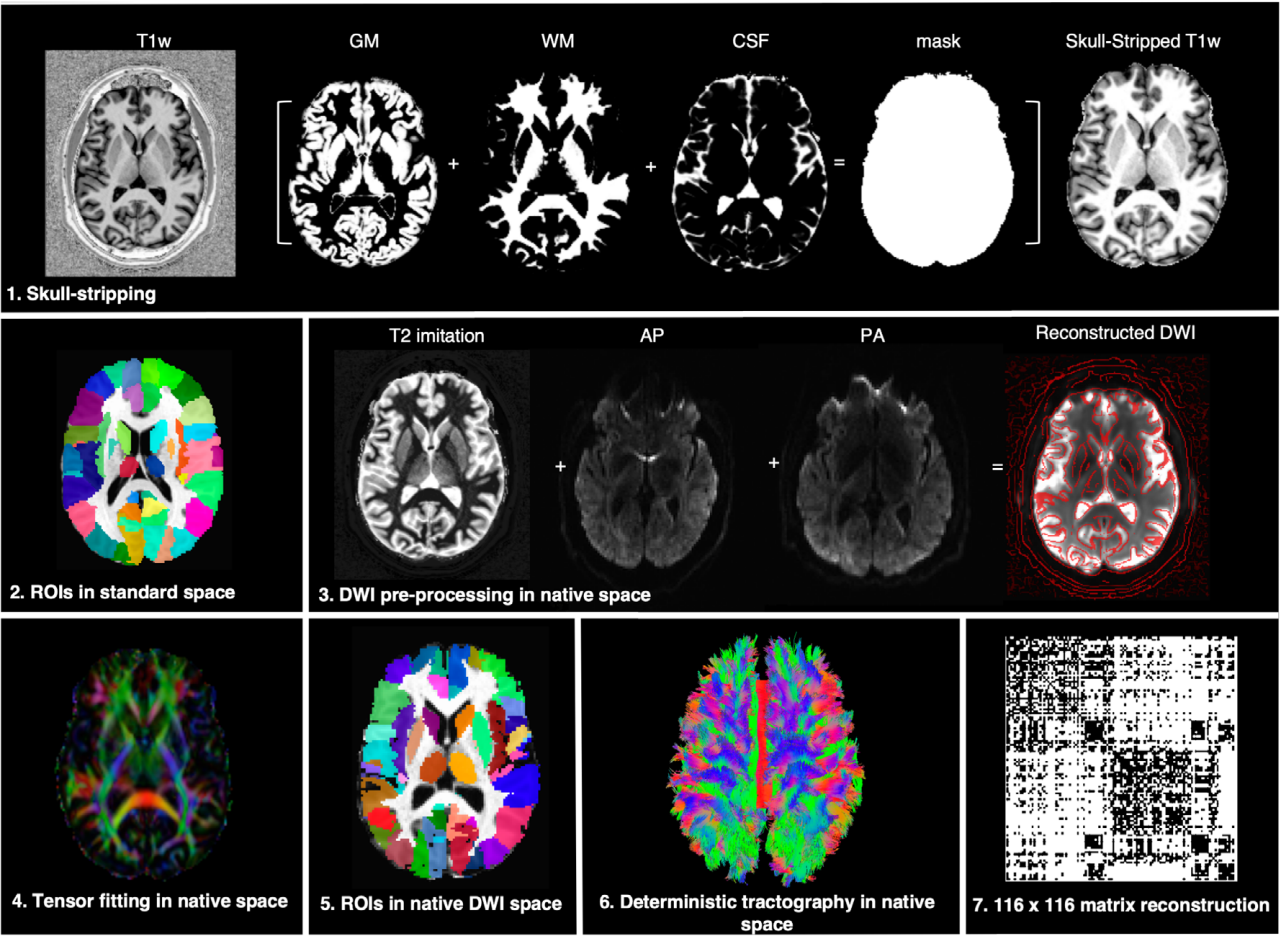
An overview of the imaging processing pipeline. (1) The uniform T1‐weighted image was skull‐stripped by segmenting gray matter (GM), white matter (WM), and cerebrospinal fluid (CSF), multiplying the resulting whole‐brain mask to the image; (2) the anatomical scan was transformed to match the AFNI standard reference template in Talairach‐Tournoux space; (3) motion and eddy current distortions were corrected for both the anterior–posterior (AP) and posterior–anterior (PA) diffusion‐weighted imaging (DWI) data sets using a T2‐weighted imitation image. AP and PA were combined to generate the final DWI reconstruction; (4) the diffusion ellipsoids and parameters were calculated using a nonlinear fitting method; (5) whole‐brain coverage with 116 anatomical regions of interest (ROIs) based on the macrolabel atlas was transferred into individual DWI space; (6) deterministic fiber tracking was performed in the native space between all pairs of ROIs; (7) a 116 × 116 adjacency matrix for each subject was created based on the normalized number of streamlines, thresholded, and used as the measure of structural pairwise connectivity between all ROI pairs (i.e., the nodes of the network)

Data preprocessing was performed using a combination of SPM12, FSL, TORTOISE, and AFNI software packages (Figure 1). DWI data were corrected for motion, eddy current distortions and susceptibility‐induced artifacts in both AP and PA directions. The AP and PA datasets were then combined using geometric averaging to generate the final corrected DWI dataset (Irfanoglu et al., 2015), following which the diffusion ellipsoids and parameters were calculated with a nonlinear fitting method (Taylor & Saad, 2013). The uncertainty estimates were calculated using jackknife resampling. Next, the individual T1‐weighted images were skull‐stripped, normalized to the standard template, and segmented into 116 anatomical regions of interest (ROIs) based on the macrolabel atlas (Eickhoff et al., 2005). In each subject, the T1‐weighted image and ROIs were registered to the individual DWI scan in its native space for proper alignment between the datasets (Greene, Cieslak, & Grafton, 2018) using the fat_proc_map_to_dti program of AFNI software. For this, a 12 degrees‐of‐freedom affine transformation matrix between the anatomical scan and the DWI reference image was calculated and applied to the T1‐weighted image and ROIs.

Consistent with the previous studies that showed the advantage of deterministic over probabilistic tractography in minimizing the negative impact of false positives during fiber reconstruction for graph theoretical analysis (Sarwar, Ramamohanarao, & Zalesky, 2019; Zalesky et al., 2016), we performed deterministic fiber tracking using the 3dTrackID program of AFNI software. The following parameters were set: fractional anisotropy threshold of 0.2, maximum propagation turning angle of 120°, minimum physical tract length of 4 mm, 8‐seed points per voxel, and the AND‐logic to determine the number of streamlines between all pairs of ROIs.

The 116 × 116 adjacency matrix was created for each subject using the normalized number of streamlines (calculated as the ratio of the number of streamlines and the number of voxels in the target mask) to account for between‐group gray matter volumetric differences in the study cohorts (Bianchi et al., 2019). The density of each individual network was proportionally thresholded to 50%, which was achieved by removing edges based on their value relative to the maximum edge weight across all networks, beginning with the weakest links. To further correct for residual, potentially spurious connections, the bottom 10% of weakest streamlines was removed in each individual network. The final group‐averaged network density was 29% in healthy controls, 30% in LD patients, and 29% in WC patients. These network densities were consistent with the previous estimates of approximately 30% connection density in the mammalian brain (Buchanan et al., 2020; Hagmann et al., 2008; Oh et al., 2014; Roberts, Perry, Roberts, Mitchell, & Breakspear, 2017). There were no significant differences in between‐group network densities (two‐sample *t* tests, all *p* ≤ .47). Network thresholding and computations of all network measures, as described below, were performed using the Brain Connectivity Toolbox.

### 3.3 Statistical analyses

Consistent with the recent studies of functional connectome in dystonia (Battistella et al., 2017; Fuertinger & Simonyan, 2018), we used the following graph theoretical measures to examine the different levels of structural network organization: network integration (characteristic path length and global efficiency), network segregation (clustering coefficient and modular organization), and nodal influence (nodal degree, strength, betweenness centrality, and hub formation).

#### 3.3.1 Network integration

The measures of characteristic path length and global efficiency were used to determine the ability of the structural network to efficiently integrate information from distributed brain regions (Sporns, 2013). The characteristic path length is computed as the average shortest path between all pairs of nodes. Inversely related to this measure is global efficiency, that is the average inverse shortest path length in the network (Rubinov & Sporns, 2010). The values from both measures were normalized with the total weights (Cheng et al., 2012).

#### 3.3.2 Network segregation

The measures of clustering coefficient and network modularity were used to examine the network capacity for specialized processing within interconnected brain regions (Sporns, 2013). The clustering coefficient is calculated as the likelihood of neighboring nodes to form segregated groups of nodes, also normalized with the total weights.

Between‐group statistical differences were determined using two‐way analysis of variance (ANOVA) with two factors: subject groups (LD, WC, controls) and examined graph measures (characteristic path length, global efficiency, clustering coefficient) at overall significance of *p* ≤ .05. If the overall group effect or its interaction with the graph measure was statistically significant, the follow‐up post hoc univariate *F*‐tests were computed to determine the differences between the groups.

Another commonly used graph measure is network modularity, which is a data‐driven approach computed without an a priori set number of network decomposition modules. Modules are defined as segregated neural communities with dense inter‐modular and weak intramodular connections. We used a multi‐iterative (*n* = 100) generalization of the Louvain community detection algorithm (Blondel, Guillaume, Lambiotte, & Lefebvre, 2008), which subdivided the network into non‐overlapping groups of nodes by maximizing the number of within‐group edges and minimizing the number of between‐group edges. The modularity of the network (*Q*) approaching the maximum value of *Q* = 1 is considered to have a strong community structure, while values of *Q* = 0–0.3 are considered to represent a random network (Newman & Girvan, 2004).

#### 3.3.3 Nodal influence and the formation of hubs

To assess the nodal influence within the network, we computed the measures of nodal degree, strength, and betweenness centrality (Rubinov, Sporns, van Leeuwen, & Breakspear, 2009). Nodal degree (*k*i) is calculated as the number of links connected to the node, and nodal strength (*s*i) is assessed as the sum of weights of links connected to the node. Betweenness centrality (*b*i) is the fraction of all shortest paths in the network that pass through a given edge, which is computed by converting the weighted connection matrix to a connection‐length matrix and normalizing it using the factor (*n* − 2)(*n* − 1). This measure reflects the probability of information transfer through a given node along the shortest path between two random nodes (Brandes, 2001). As such, edges with higher values suggest participation in a large number of shortest paths and are of higher importance for controlling the information flow. Between‐group statistical differences in nodal degree, strength, and betweenness centrality were determined using nonparametric permutation tests with 10,000 iterations at *p* ≤ .016 to correct for multiple comparisons (Nichols & Holmes, 2002).

Network hubs were determined based on nodal degree and strength of at least one *SD* greater than the average total degree and strength of the group network. The hubs were classified into provincial (i.e., linking nodes within a module with PI = 0.3–0.75) and connector (i.e., linking nodes between the modules with PI ≤ 0.3) (van den Heuvel & Sporns, 2011).

#### 3.3.4 Clinical correlates of network alterations

The relationship between clinical features of LD and WC, as described above and Table 1, with significantly abnormal network measures was assessed using Spearman's rank correlation coefficients at *p* ≤ .05.

#### 3.3.5 Potential methodological limitations

A general challenge of studies using graph theoretical analysis is the availability of a variety of methodological choices, which are being continuously adapted based on the goals of the study rather than the established guidelines (Maier‐Hein et al., 2017). For example, there is no consensus on the choice of connectivity metrics (e.g., widely used number of streamlines vs. alternative measures of fractional anisotropy) (de Brito Robalo et al., 2020), network reconstruction (e.g., deterministic vs. probabilistic tractography) (Sarwar et al., 2019; Zalesky et al., 2016), network thresholding (e.g., proportional vs. consistency) (Buchanan et al., 2020), or the choice of atlases for selection of regions of interest (Wei, Cieslak, Greene, Grafton, & Carlson, 2018). Similarly, there is a range of statistical tests (e.g., parametric vs. permutation) (Nichols & Holmes, 2002; Veronese et al., 2019) and methods for multiple comparison corrections (e.g., false discovery rate vs. family‐wise error) (Chen, Lu, & Yan, 2018; Turkheimer, Pettigrew, Sokoloff, Smith, & Schmidt, 2000) that can be applied to graph theoretical measures. A series of methodological studies are warranted to optimize these parameters based on large‐scale empirical or simulated data in order to develop standard recommendations.

## 4 RESULTS

Regional alterations in white matter integrity using tract‐based spatial statistics, gray matter organization using voxel‐based morphometry and cortical thickness analysis, as well as resting‐state functional connectivity using independent component and graph theoretical analyses in LD and WC patients were reported in our previous studies (Battistella et al., 2017; Bianchi et al., 2019; Fuertinger & Simonyan, 2017; Fuertinger & Simonyan, 2018).

**FIGURE 2:**
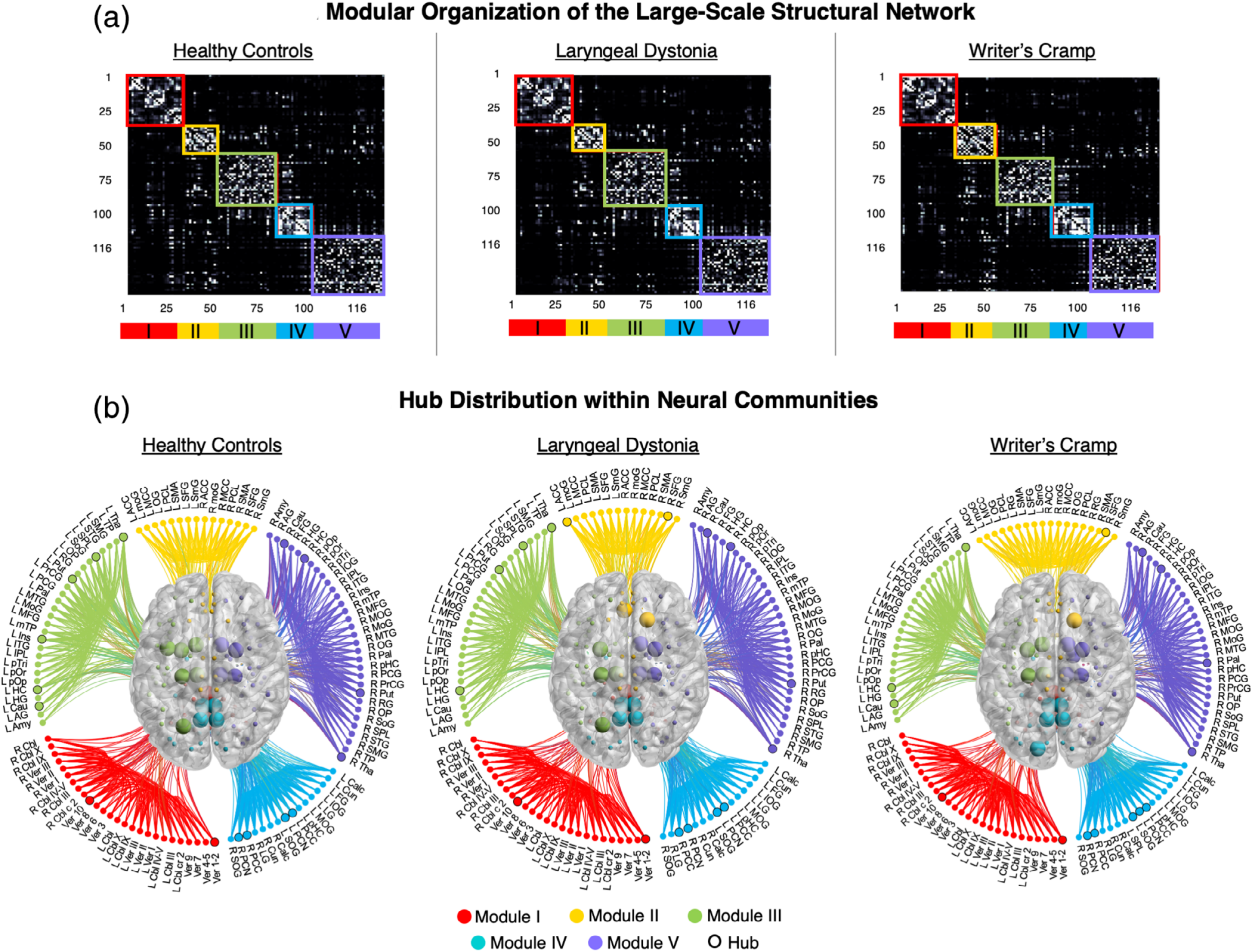
Overall structural neural community architecture. (a) The 116 × 116 matrices show averaged number of streamlines between each pair of brain regions in healthy controls, patients with laryngeal dystonia and writer's cramp, respectively. The neural community partition is based on the normalized number of streamlines between each pair of regions. The modules of the network are ordered and visualized according to the community structure. (b) The connectograms shows the same modules as in (a), with nodes (circles) labeled according to their regions and the degree/strength hubs (larger circles, bold labels) distributed within each module. 3D brain view in the center of the connectograms shows the spatial distribution of neural communities, with spheres representing hubs in each module. The modules are color‐coded as follows: red—Module I, yellow—Module II, green—Module III, blue—Module IV, purple—Module V. Results were visualized with BrainNet Viewer, NeuroMArVL, and MATLAB scripts. ACC, anterior cingulate cortex; AG, angular gyrus; Amy, amygdala; Calc, calcarine gyrus; Cau, caudate nucleus; Cbl, cerebellum; Cun, cuneus; FG, fusiform gyrus; Hip, hippocampus; HG, Heschl's gyrus; Ins, insula; IOG, inferior occipital gyrus; IPL, inferior parietal lobule; ITG, inferior temp gyrus; LG, lingual gyrus; MCC, middle cingulate; MFG, middle frontal gyrus; moG, middle orbital gyrus; MOG, middle occipital gyrus; MoG, medial orbital gyrus; MTG, middle temporal gyrus; mTP, medial temp pole; OG, olfactory gyrus; OP, operculum; Pal, pallidum; PCG, postcentral gyrus; PCC, posterior cingulate cortex; PCL, paracentral lobule; PCN, precuneus; pHC, parahippocampal; pOp, pars opercularis; pOr, pars orbitalis; PrCG, precentral gyrus; pTri, pars triangularis; Put, putamen; RG, rectal gyrus; SFG, superior frontal gyrus; SMA, supplementary motor area; SmG, superior medial gyrus; SMG, supramarginal gyrus; SOG, superior occipital gyrus; SoG, superior orbital gyrus; SPL, superior parietal lobule; STG, superior temporal gyrus; Tha, thalamus; TP, temporal pole; Ver, cerebellar vermis

The overall large‐scale structural architecture was comparable between the dystonic and healthy states, forming five different neural communities (modules) (Figure 2a). No statistically significant differences were found in global efficiency, characteristic path length, or clustering coefficient between TSFD patients and healthy controls (ANOVA: group *F*_2,45_ = 0.64, *p* = .83; group × graph measure interaction *F*_4,90_ = 0.13, *p* = .97).

However, the network modular organization in TSFD patients was altered compared to healthy controls due to the abnormal nodal assignment to neural communities. Specifically, LD patients showed the shrinkage of module II by 7% due to the loss of left olfactory gyrus, which relocated into the expanded module III (4% gain) (Figure 2b). Conversely, WC patients compared to healthy controls had the expansion of module II by 20% due to the gain of bilateral rectal gyrus from modules III and V as well as the expansion of module IV by 6% due to the gain of left superior parietal lobe from module III (Figure 2b).

**FIGURE 3:**
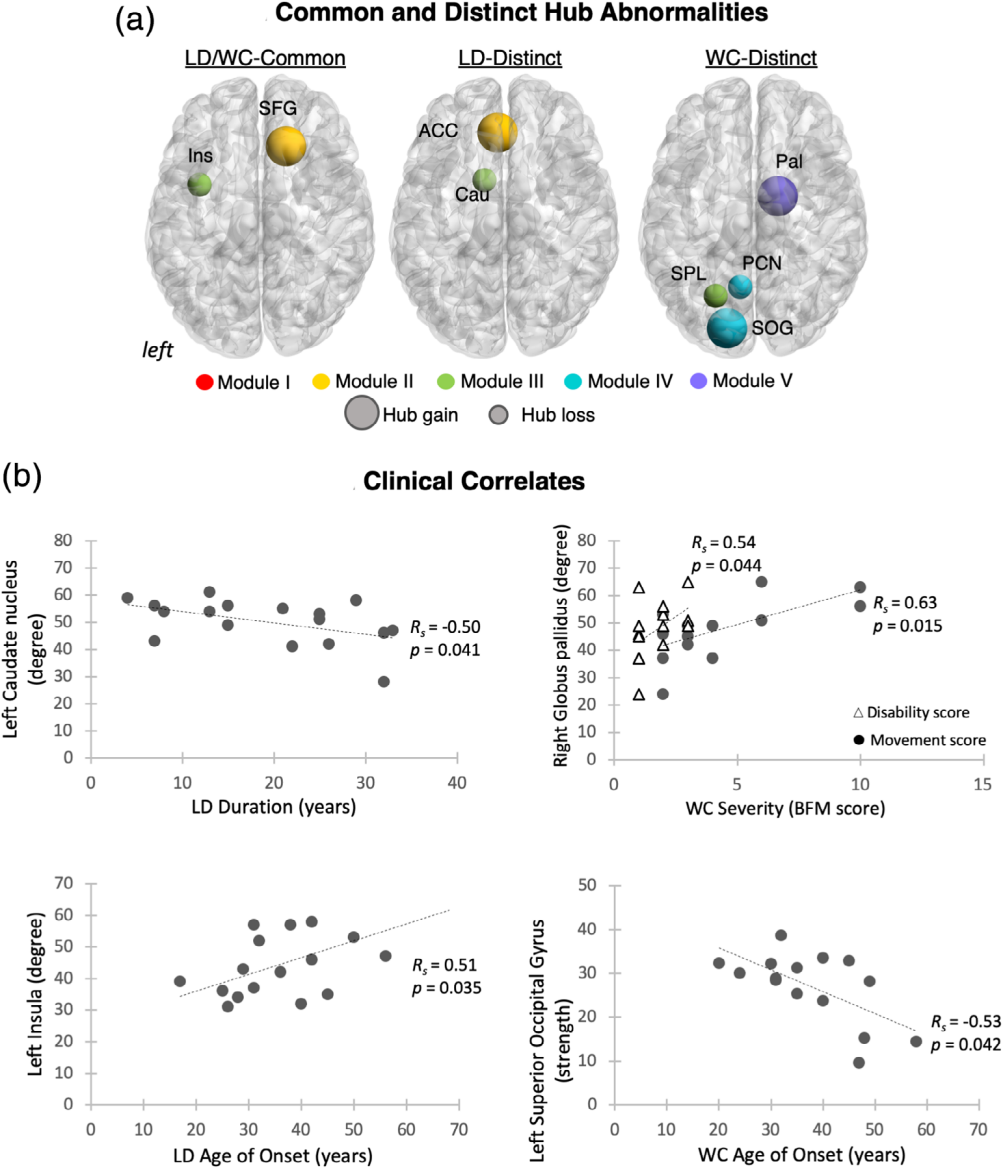
Hub abnormalities and clinical correlates of network‐wide alterations. (a) The 3D brain views show common and distinct abnormalities in hub formation in patients with laryngeal dystonia and writer's cramp compared to healthy controls, respectively. Larger circles depict hubs gained within the respective module; smaller circles represent hubs lost within the respective module; hubs are color‐coded based on their modular affiliation. (b) The scatterplot shows the correlation between the clinical characteristics of dystonia and altered nodal measures within the respective networks. The duration and age of dystonia onset were established as part of neurological/laryngological evaluation; dystonia severity was assessed using the Burke–Fahn–Marsden Dystonia Rating Scale (BFM), including the movement and disability scores

Changes in network modular structure of TSFD patients were further instigated by abnormal hub formation. Based on nodal degree, both TSFD patients and healthy controls shared hubs in the right caudate nucleus, bilateral hippocampus, precuneus, putamen, and thalamus (Table 2). Based on nodal strength, all patients and controls shared hubs in the right precuneus, bilateral putamen, thalamus, posterior cingulate cortex, and cerebellar vermis (Table 2). Notably, the right posterior cingulate hub was downgraded from its connector status in healthy controls to the provincial status in both LD and WC patients. In addition, hubs in the right putamen and left posterior cingulate cortex were downgraded from their connector influence in healthy controls to provincial influence in WC patients, thus affecting the network information flow passing through these regions. Compared to healthy controls, both LD and WC patients commonly lost the left insular hub but gained the right superior frontal gyrus as degree connector hub (Figure 3a, Table 2).

**TABLE 2:** Shared and distinct hubs based on nodal strength and degree in the group‐averaged structural networks

	Degree			Strength		
Brain region	Healthy controls	Laryngeal dystonia	Writer's cramp	Healthy controls	Laryngeal dystonia	Writer's cramp
*Shared hubs in healthy controls and patients with laryngeal dystonia and writer's cramp*						
R caudate nucleus—M‐V	**60.2**	**57.9**	**57.1**			
L hippocampus—M‐III	**50.1**	**53.8**	**52.7**			
R hippocampus—M‐V	**55.4**	**53.9**	**54.9**			
L Precuneus—M‐IV	**57.6**	**58.3**	**51.7**			
R Precuneus—M‐IV	**59.6**	**57.7**	**51.9**	**41.1**	**36.8**	**38.6**
L putamen—M‐III	**64.3**	**68.9**	**64.9**	**39.6**	**43.4**	**42.7**
R putamen—M‐V	**65.8**	**73.0**	*67.1*	**44.5**	**47.6**	*46.1*
L thalamus—M‐III	**54.8**	**58.8**	**54.5**	**35.2**	**34.0**	**33.4**
R thalamus—M‐V	**53.6**	**57.4**	**55.9**	**36.1**	**36.9**	**33.8**
Cerebellar vermis 1–2—M‐I				*56.9*	*60.0*	*68.5*
Cerebellar vermis 10—M‐I				*50.9*	*53.9*	*64.7*
L posterior cingulate cortex—M‐IV				**53.9**	**52.4**	*51.4*
R posterior cingulate cortex—M‐III				**57.6**	*56.4*	*54.7*
*Commonly lost hub in patients with laryngeal dystonia and Writer's cramp*						
L insula—M‐III	**51.3**	45.3	40.3			
*Commonly gained hub in patients with laryngeal dystonia and Writer's cramp*						
R superior frontal gyrus—M‐II	47.3	**55.5**	**48.9**			
*Distinctly gained hub in patients with laryngeal dystonia*						
L anterior cingulate cortex—M‐II				30.4	**35.1**	26.8
*Distinctly lost hub in patients with laryngeal dystonia*						
L caudate nucleus—M‐III	**53.3**	50.2	**54.5**			
*Distinctly gained hubs in patients with Writer's cramp*						
R pallidum—M‐V	44.4	48.1	**48.5**			
L superior occipital gyrus—M‐IV				30.9	30.2	*35.0*
*Distinctly lost hubs in patients with Writer's cramp*						
L superior parietal lobule—M‐V	**49.3**	**51.0**	44.1			
L Precuneus—M‐IV				**35.9**	**34.5**	32.2
Network mean ± *SD*	33.9 ± 14.8	35.1 ± 15.1	33.0 ± 14.0	17.4 ± 15.0	17.2 ± 14.7	17.2 ± 15.9

*Note*: Italic values are provincial hubs (PI ≤ 0.3) and bold values are connector hubs (PI 0.3–0.75).

Abbreviations: L, left; M, network module affiliation of a given hub; PI, participation index; R, right.

TSFD‐form specific hub alterations were as follows. LD patients distinctly gained the left anterior cingulate cortex (ACC) as strength connector hub and lost the left caudate nucleus as degree connector hub compared to healthy controls and WC patients (Figure 3a, Table 2). On the other hand, the WC connectome distinctly gained the right pallidum as degree connector hub and the left superior occipital gyrus (SOG) as strength provincial hub, while losing the left superior parietal lobule as degree connector hub and the left precuneus as strength connector hub (Figure 3a, Table 2). Thus, alterations of the TSFD structural connectome were characterized by abnormal nodal migration across the neural communities and both common and distinct patterns of abnormal hub formation within these communities in LD and WC patients.

**FIGURE 4:**
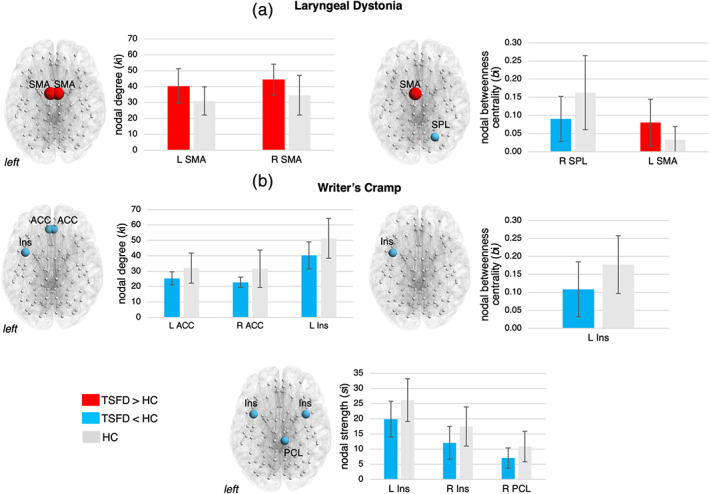
Significant regional alterations of the structural connectome of dystonia patients compared to healthy controls. 3D brain renderings and bar graphs show significant regional changes in nodal degree, nodal strength, and betweenness centrality in (a) patients with laryngeal dystonia and (b) patients with writer's cramp compared to healthy controls, respectively. Error bars show *SD*; red bars indicate increases in nodal measures in patients compared to controls; blue bars depict decreases in nodal measures in patients compared controls; gray bars show the normative values in healthy controls. ACC, anterior cingulate cortex; HC, healthy controls; Ins, insula; L, left; PCL, paracentral lobule; R, right; SMA, supplementary motor area; SPL, superior parietal lobule; TSFD, task‐specific focal dystonia

At the regional level, structural networks in both forms of TSFD showed significant alterations in nodal degree, strength, and betweenness centrality compared to healthy controls (Figure 4, Table 3). LD connectome was characterized by increased nodal degree and betweenness centrality in the supplementary motor area (SMA) and decreased betweenness centrally in the right superior parietal lobule (all *p* ≤ .016) (Figure 4a, Table 3). Conversely, WC patients showed decreased measures of nodal degree, strength or betweenness centrality in the bilateral ACC, insula, and right paracentral lobule (all *p* ≤ .016) (Figure 4b, Table 3). Thus, network nodes were distinctly altered in LD and WC patients compared to healthy controls, further pointing to dystonia‐form specific neural changes.

**TABLE 3:** Differences in local graph metrics between TSFD patients and healthy controls

Brain region	Laryngeal dystonia	Healthy controls		
	*Nodal degree*		*SDS*	*p‐value*
L supplementary motor area	40.35	30.94	9.42	0.009
R supplementary motor area	44.41	34.56	9.85	0.016
	*Betweenness centrality*		*SDS*	*p‐value*
L supplementary motor area	0.08	0.03	0.05	0.009
R superior parietal lobule	0.09	0.16	−0.07	0.014
	**Writer's cramp**	**Healthy controls**		
	*Nodal degree*		*SDS*	*p‐value*
L insula	40.33	51.31	−1.98	.009
L anterior cingulate cortex	25.33	32.06	−6.73	.016
R anterior cingulate cortex	22.80	31.63	−8.83	.008
	*Nodal strength*		*SDS*	*p‐value*
L insula	19.89	26.13	−6.23	.010
R insula	12.07	17.47	−5.40	.014
R paracentral lobule	7.07	10.84	−3.78	.016
	*Betweenness centrality*		*SDS*	*p‐value*
L insula	0.11	0.18	−.07	.014

Abbreviations: L, left; R, right; SDS, standard difference score.

#### 4.1.1. Clinical correlates of network alterations

There were no significant relationships between the duration of dystonia and the severity of either WC (*p* ≥ .23) or LD (all *p* ≥ .24), as well as between the age of LD onset and its severity as assessed by BFM movement and disability scores (*p* ≥ .21). The age of WC onset showed a significant negative correlation with the BFM disability score (*R*_*s*_ = −0.76, *p* = .002) but not the BFM movement score (*p* = .47).

In addition, significant correlations were found between abnormal nodal degree of the left caudate nucleus and LD duration (*R*_*s*_ = −0.50, *p* = .041) and nodal degree of the left insula and LD age of onset (*R*_*s*_ = 0.51, *p* = .035) (Figure 3b). In WC, the symptom severity significantly correlated with abnormal nodal degree of the right globus pallidus (disability score: *R*_*s*_ = 0.5, *p* = .04; movement score: *R*_*s*_ = 0.63, *p* = .015), whereas the age of dystonia onset showed a correlation with abnormal nodal strength of the left SOG (*R*_*s*_ = 0.51, *p* = .035) (Figure 3b).

## 5 DISCUSSION

Dystonia has been long considered a basal ganglia disorder (Berardelli et al., 1998; Defazio, Berardelli, & Hallett, 2007), with recent evidence suggesting the presence of additional abnormalities in the function of higher‐order sensorimotor and associative cortical areas, especially in patients with TSFDs, such as LD and WC (Battistella et al., 2017; Fuertinger & Simonyan, 2018; Gallea, Horovitz, Ali Najee‐Ullah, & Hallett, 2016). Mechanistic alterations of the functional connectome in these dystonias have been demonstrated to involve a top‐down disruption of the sensorimotor network due to hyperexcitable parietal‐basal ganglia connectivity (Battistella & Simonyan, 2019) and abnormal increases of striatal dopamine release contributing to the altered balance between the direct and indirect basal ganglia pathways during production of dystonic behaviors (Berman, Herscovitch, Hallett, & Simonyan, 2010; Simonyan, Berman, Herscovitch, & Hallett, 2013). The present study demonstrates that, in addition to profoundly altered functional network in focal dystonia, its structural connectome is characterized by large‐scale aberrations. Overall, the global configuration of the structural connectome was altered due to abnormal transfer of prefrontal and parietal nodes between neural communities and the reorganization of normal hub architecture, involving commonly lost hub in the left insula and commonly gained hub in the superior frontal gyrus in both LD and WC patients compared to healthy controls. Other prominent common changes of the TSFD structural connectome involved the basal ganglia, parietal and cingulate cortical regions, whereas premotor (SMA) and occipital (SOG) abnormalities distinguished between LD and WC, respectively.

The loss of insular hub in the LD and WC structural connectomes is in line with a similar deficit found in the functional connectome of these patients (Battistella et al., 2017). Other neuroimaging studies reported abnormal activity during speaking in LD and writing in WC (Ali et al., 2006; Ceballos‐Baumann, Sheean, Passingham, Marsden, & Brooks, 1997; Lerner et al., 2004; Peller et al., 2006; Simonyan & Ludlow, 2010), decreased cortical thickness linked to distinct clinical phenotypes of LD (Bianchi et al., 2017), and changes in GABA_A_ receptor density in WC (Gallea et al., 2018; Peller et al., 2006). The insula is an important cortical outflow hub, being involved in various cognitive and sensorimotor behaviors, including generation of internal representations of intended movements (Karnath, Baier, & Nagele, 2005; Menon & Uddin, 2010; Sridharan, Levitin, & Menon, 2008). Our finding of the loss of the insula as network connector hub in both LD and WC, as well a significant correlation between its abnormal connectivity and LD age of onset, may suggest the failure of this region to coordinate the information flow between neural communities that participate in the control of sensorimotor processing during movement planning. Furthermore, together with the hub emergence in the SFG and nodal changes in the SMA and ACC, abnormal insular participation within the network may reflect abnormal monitoring of internal movement representations, decision making and working memory during performance of dystonia‐affected behaviors (Bush et al., 2002; Bush, Luu, & Posner, 2000; Daw, O'Doherty, Dayan, Seymour, & Dolan, 2006; Kovach et al., 2012; Pochon et al., 2002; Xu et al., 2013; Zeuner et al., 2016).

Commonly altered hub formation in the basal ganglia may play an important role in further facilitation of abnormal traffic within LD and WC structural networks. Specifically, significant relationships between decreased connectivity of the caudate nucleus and LD duration as well as increased connectivity of the globus pallidus and WC severity suggest that these regions may take part in abnormal control of goal‐directed motor behaviors and altered suppression of error feedback monitoring (Redgrave et al., 2010). It is important to note that the globus pallidus is currently defined as an effective deep brain stimulation (DBS) site in patients with dystonia (Volkmann et al., 2012). While the mechanisms of its neuromodulatory effects remain unclear, it is possible that the therapeutic outcome of pallidal DBS might, in part, be due to corrective reversal of altered pallidal connectivity.

Other regions that were commonly altered in the LD and WC connectomes were the cingulate and parietal cortical areas. Changes in structural connectivity of the cingulate cortex points to the aberrant motor action selection and error correction (Arrighi et al., 2016; Holroyd & Coles, 2002) prior to the output of a dystonic behavior by the primary motor cortex. Alterations of the structural and functional organization of the parietal cortex have been recently discussed as important contributors to the dystonia pathophysiology, being linked to the polygenic and extrinsic risks for disorder development (de Lima Xavier & Simonyan, 2019; Putzel et al., 2018). With the focus on the sensorimotor control of highly learned motor behaviors, such as speaking and writing, failure of these network nodes in TSFD patients suggests a breakdown in processing and integration of sensorimotor information at the highest levels of structural network connectivity.

The connectomes in each form of focal dystonia were further characterized by a set of distinct alterations of hubs and nodes. Regional abnormalities in the LD structural network most prominently involved the SMA, which is known to control action preparation, initiation and selection (Bonini et al., 2014; Swann et al., 2012) during speech production (Fuertinger, Horwitz, & Simonyan, 2015). The SMA establishes direct structural projections with the laryngeal motor cortex, is functionally active during preparation and production of various voluntary laryngeal tasks, and partakes in the preparatory phase of vocal motor command execution, syllable sequence production, and speech error detection (Gauvin, De Baene, Brass, & Hartsuiker, 2016; Loucks, Poletto, Simonyan, Reynolds, & Ludlow, 2007; Rong, Isenberg, Sun, & Hickok, 2018; Simonyan & Jurgens, 2002). Its abnormally increased involvement within the LD structural network suggests the likely presence of a compensatory overload at the preparatory phases of speech motor execution forged, in part, by dystonic activity of the motor cortex. Thus, LD‐specific changes of the structural connectome were centered around the altered consolidation of sensorimotor information due to abnormal motor preparatory function, which is necessary for the proper execution of speech motor commands.

Distinct alterations of the WC connectome involved abnormal SOG hub formation that was not present in healthy controls. Although a rather novel concept for the dystonia pathophysiology, the emergence of network disruptions involving the occipital region is in agreement with several of previous studies. A systematic review of post‐stroke movement disorders found that 70% of dystonia‐causing lesions occur in the occipital lobe (Suri et al., 2018). While secondary dystonias differ from isolated task‐specific dystonia, such as LD and WC, in their causative mechanisms, lesion studies have traditionally provided important insights into causative brain function (Adolphs, 2016), including the significance of the basal ganglia in dystonia pathophysiology (Marsden, Obeso, Zarranz, & Lang, 1985). As such, similarities in brain alterations between secondary and isolated dystonias may point toward common underlying pathways involved in the occurrence of dystonic symptoms in general. More recently, another study in patients with focal dystonia, including WC, demonstrated that occipital regions contribute to the formation of aberrant network kernel (Fuertinger & Simonyan, 2018) and, together with premotor and parietal regions, support processing of visual temporal discriminatory stimuli in TSFD patients (Maguire, Reilly, & Simonyan, 2020). Future studies are warranted to conduct a detailed investigation of the involvement of the occipital region in the pathophysiology of dystonia.

The absence of graph measure abnormalities in the primary motor cortex and cerebellum may seem at first a counterintuitive finding given the fact that TSFDs are movement disorders. Notably, graph analysis is a data‐driven methodology applied to the whole‐brain data versus data‐driven methods applied to a given region or network as in case of region‐of‐interest or seed‐based studies described in the previous reports, which defined the presence of functional and structural alterations in these regions (Neychev et al., 2011; Simonyan, 2018; Zoons et al., 2011). Our current findings suggest that, at the level of a whole‐brain network, other brain regions that are involved in the control of movement planning, preparation, and integration of sensorimotor information may play a more prominent role in the formation of the TSFD structural connectome than the primary motor cortex and cerebellum. This finding is in line with our recent study, which showed that functional alterations in premotor‐parietal‐basal ganglia circuitry precede those in the primary motor cortex, and the network disruption likely occurs well before the dystonic behavior is produced by the primary motor cortex (Battistella & Simonyan, 2019).

In conclusion, our data provide new evidence of abnormal large‐scale structural architecture in focal dystonia and propose that TSFD is a network disorder at *both* structural and functional levels. As several studies have suggested that non‐invasive neuromodulation approaches, such as repetitive transcranial magnetic stimulation and transcranial direct current stimulation, modulate brain networks rather than only local targets of stimulation (To, De Ridder, Hart Jr., & Vanneste, 2018), the detailed knowledge of large‐scale network organization in dystonia may prove useful in defining novel targets for therapeutic neuromodulation in this disorder.

## CONFLICT OF INTEREST

The authors declare no conflicts of interest.

## Data Availability

Upon the acceptance of this manuscript, the research data used in this study will be archived in the figshare public repository. Analytic codes used in this study are publicly available at https://simonyanlab.hms.harvard.edu/resources.
